# An underestimated pitfall of oral candidiasis in head and neck cancer patients undergoing radiotherapy: an observation study

**DOI:** 10.1186/s12903-021-01721-x

**Published:** 2021-07-16

**Authors:** Imjai Chitapanarux, Somying Wongsrita, Patumrat Sripan, Panithan Kongsupapsiri, Panchalee Phakoetsuk, Siriarrayapa Chachvarat, Kittikun Kittidachanan

**Affiliations:** 1 Department of Radiology, Faculty of Medicine, Chiang Mai University, Chiang Mai, Thailand; 2 Northern Thai Research Group of Radiation Oncology (NTRG-RO), Faculty of Medicine, Chiang Mai University, Chiang Mai, Thailand; 3 Division of Radiation Oncology, Maharaj Nakornratchasima Hospital, Nakornratchasima, Thailand; 4 Research Institute for Health Sciences, Chiang Mai University, Chiang Mai, Thailand; 5 Nakornping Cancer Center, Nakornping Hospital, Chiang Mai, Thailand; 6 Maharaj Nakhon Si Thammarat Hospital, Nakhon Si Thammarat, Thailand

**Keywords:** Candidiasis, Radiotherapy, Chemotherapy, Head and neck cancer, Accuracy

## Abstract

**Background:**

Oral candidiasis is a common problem associated with head and neck radiation therapy (RT) consequences being pain, burning sensation, taste change, and systemic infection. There are difficulties in differentiating between oral candidiasis and radiation induced oral mucositis. We conducted a prospective study to explore the incidence of clinical oral candidiasis and evaluate the accuracy of diagnosis among head and neck cancer (HNC) patients receiving RT or concurrent chemoradiotherapy (CCRT).

**Methods:**

This study included 86 HNC patients who had no clinical signs or symptoms of oral candidiasis. Oral mucosa and tongue swabs were carried out and analyzed three times by fungal cultures at the following time points: (1) before RT, (2) at the time of clinically diagnosed candidiasis or during RT at between the 15th–17th fraction (whichever occurred first), and (3) at the end of RT. The accuracy of clinical oral candidiasis was explored and confirmed by fungal colonization techniques. The incidence and risk factors associated with fungal colonization before and throughout the treatment were analyzed.

**Results:**

The overall incidence of clinical oral candidiasis was 53.5% throughout the course of RT. Confirmation of fungal colonization was found in 39.5%, 65.9%, and 57.7% of cases before RT, during RT, and end of RT, respectively. The accuracy of the diagnosis using only clinical presentation was demonstrated in 60%, 50%, and 52% before RT, during RT, and end of RT, respectively. Logistic regression analysis showed that age, CCRT, and using 2% viscous lidocaine solution were independent risk factors for fungal colonization.

**Conclusions:**

The results of this study demonstrated an underestimation of clinical oral candidiasis before and throughout the course of radiotherapy from using only clinical sign and symptoms. Crucial attention to detail and testing in the oral examination could improve decision making for detection of oral candidiasis in HNC patients receiving RT or CCRT.

## Background

In Thailand, the incidence of Head and Neck cancer (HNC) is rising with age-standardized incidence rates (ASRs) of 14.2 per 100,000 for males and 9.7 per 100,000 for females in 2007–2009 compared to 15.7 per 100,000 for males and 10.7 per 100,000 for females in 2010–2012 [1]. GLOBOCAN 2020 reported 11,351 new HNC cases, both sexes and all ages [2]

In Thailand, the incidence of Head and Neck cancer (HNC) is rising with age-standardized incidence rates (ASRs) of 14.2 per 100,000 for males and 9.7 per 100,000 for females in 2007–2009 compared to 15.7 per 100,000 for males and 10.7 per 100,000 for females in 2010–2012 [3]. Oral mucosal colonization has been discovered in between 90 and100% of irradiated HNC patients with candida infection being found most commonly, ranging from 17 to 52% [4–8]. Many studies observed oral fungal colonization and infection increased during RT [5–7]. The risk factors for colonization and infection are still controversial in this group of patients. Age, denture wearing, smoking, alcohol drinking, and xerostomia were identified as the potential determinant factors [3, 5, 8, 9].

There are multiple clinical presentations of oral candidiasis. Clinical oral candidiasis was and is diagnosed by the following sign and symptoms: the presence of pseudomembranous (white oral thrush), redness of the mucosa, oral pain, and burning sensation in the oral cavity. These signs and symptoms still used in diagnosis today but there are some difficulties in distinguishing oral candidiasis from radiation induced oral mucositis [10, 11]. Oral pain is the main symptom both conditions. This symptom is well documented as being significantly troublesome to irradiated HNC patients due to the chronic nature of the pain or inconvenience during mastication, resulting in malnutrition [4, 6, 12, 13]. Pseudomembranous candidiasis is the most common manifestation with scrapable white plaque [12–14], while the appearance of erythematous candidiasis is rare [13, 14].

This is a prospective analytical study conducted on the HNC patients undergoing RT with or without chemotherapy to investigate the accuracy of clinically diagnosed oral candidiasis. We also investigated the pattern, incidence, and the risk factors of fungal colonization before and throughout the treatment.

## Methods

### Patient and design

We enrolled all the patients with a histological diagnosis of HNC with the following inclusion criteria: age equal to or more than 18 years, underwent curative RT either definitive RT with the dose of 70 Gy or postoperative RT with the dose of 60–66 Gy depending on surgical margin and/or extracapsular extension with or without concurrent chemotherapy (weekly cisplatin 40 mg/m^2^ or weekly carboplatin 100 mg/m^2^ depending on the patient’s renal function), and RT field enclosed the oral cavity and oropharynx. The exclusion criteria were patients with a clinical diagnosis of oral candidiasis before participation in the study, were receiving any form of antifungal drugs within one week of entering this study, had trismus and could not open their mouth to facilitate performance of the oral mucosa swab, or had a history of receiving head and neck RT. All eligible patients received a complete clinical dental examination and an necessary dental procedures before starting RT. Oral hygiene was also assessed and scored with a simplified oral hygiene index (OHI-S) [15] into a final classification of excellent, good, fair, or poor status. Oral hygiene and dental care education were also provided to all patients. Using any mouthwash as typical practice was allowed in this study except for any mouthwash which contained antibiotic or antifungal agents.

Written informed consent was obtained from all patients before beginning of the study. This study was approved by the Research Ethics Committee of Faculty of Medicine, Chiang Mai University, approved on 17 September 2015 (approval number: 395/2015). All procedures performed in this study involving human participants were adhered to the ethical standards of the Declaration of Helsinki.

### Study procedures

We performed the oral mucosa and tongue swabs and analyzed fungal cultures three times at the following time points: before RT, during RT (halfway between the 15th–17th RT fraction), and after RT (immediately after completion of RT). For patients who had a clinical diagnosis of oral candidiasis, e. g. presence of pseudomembranous (white oral thrush) at any time points of treatment; redness mucosa or burning sensation in the oral cavity that occurred before the end of the 1st week of treatment (since these signs and symptoms are difficult in differentiating candida infection and acute mucositis) which usually appear in the dose range of 10–20 Gy according to Epstein JB et al. [9]. The oral mucosa, and tongue swabs were carried out promptly. All clinically diagnosed candidiasis patients immediately received antifungal agents, e.g., nystatin oral suspension 4 mL (400,000 units) four times daily, the suspension being retained in the mouth for 1–2 min then swallowed for 1 week, clotrimazole troche 10 mg per oral 4 times daily for 1 week if the patient could not tolerate the suspension form or fluconazole 100 mg once a day for 1 week if the patient could not tolerate either of the previous forms of antifungal agents.

### Diagnosis

A swab from each patient was cultured on Sabouraud’s dextrose agar. After 48 h of incubation at 27 ± 2 °C, the fungal viable counts were assessed. For identification of the species of each microorganism, we used conventional yeast identification methods, i.e., carbohydrate assimilation tests, germ tube formation, and morphology agar. Clinical evaluation and an inspection of the oral mucosa were assessed once a week during the treatment using the common terminology criteria for adverse events (CTCAE) Version 4.03. All prescription of mouthwash or 2% viscous lidocaine solution was also recorded.

### Statistical analyses

The sample size was determined by assuming the proportion of patients with oral candidiasis was 0.5, which is conservative and gives the largest sample size with a 95% confidence interval width of 10% [16]. The calculated sample size was 97 patients. Categorical variables are presented as frequencies and percentages, and continuous variables as medians and interquartile ranges (IQR). Accuracy of clinical diagnosis of oral candidiasis before RT, during RT, and end of RT were evaluated using fungal colonization as the gold standard method of diagnosis. Fisher’s exact test and Wilcoxon-Mann–Whitney tests were performed to compare the characteristics between fungal colonization and non-fungal colonization. Logistic regression was performed to identify factors associated with the incidence of fungal colonization and clinical diagnosis throughout the treatment. Variables with a *p* value < 0.25 in the univariate analysis were included in the multivariate analysis. In the two-tailed test *p* < 0.05 was considered to be significant. All analyses were performed using STATA software version 16 (Stata Corp, College Station, TX). Accuracy of clinically diagnosed oral candidiasis was calculated as a ratio of precise diagnosis (true positive + true negative) divided by all cases as shown in this formula: Accuracy = (TP + TN)/(TP + TN + FP + FN), where TP was true positive (clinical diagnosed candidiasis and positive fungal colonization), TN was true negative (no clinical diagnosed candidiasis and negative fungal colonization), FP was false positive (clinical diagnosed candidiasis but negative fungal colonization), and FN was false negative (no clinical diagnosed candidiasis but positive fungal colonization).

## Results

### Patients and characteristics

Between October 2015 and October 2017 a total of 97 patients were enrolled onto the study. However due to a period of 3–6 weeks between the enrollment date to the first date of beginning RT, we excluded 11 patients for the following reasons: two patients developed distant metastasis and the aim of treatment was changed to palliative RT, one patient had trismus at the first date of RT, three patients had a clinical diagnosis of oral candidiasis and the RT field did not cover the oropharynx or oral cavity in five patients. The study proceeded with 86 patients.

Patient and treatment characteristics are shown in Table 1. There were 59 (68.6%) male and 27 (31.4%) female patients with a median age of 60 years (IQR 56–66). Most of our patients stopped smoking and alcohol drinking before the treatment. The most frequent OHI-S index in our patient population was a good rate of 79%. Sixteen (18.6) patients were edentulous and nine (10.5%) patients wore dentures. Nasopharyngeal cancer was the most common cancer followed by oropharyngeal cancer and oral cavity cancer. All nasopharyngeal cancer patients had WHO type II non-keratinizing squamous cell carcinoma and all of the non-nasopharyngeal cancer patients had squamous cell carcinoma.

**Table 1:** Patient and treatment characteristics

Characteristic	n (%)
Sex	
Male	59 (68.6)
Female	27 (31.4)
Age	
Median (IQR)	60 (56–66)
Range	21–83
Underlying disease	
DM	5 (5.8)
HT	23 (26.7)
HIV	1 (1.2)
Personal history	
Smoking	
Never used	21 (24.4)
Quit	53 (61.6)
Use currently	12 (14)
Alcohol	
Never used	31 (36)
Quit	43 (50)
Using currently	12 (14)
Chewing betel	
Never	75 (87.2)
Quit using	5 (5.8)
Using currently	6 (7)
Oral status	
Oral hygiene	
Excellent	3 (3.5)
Good	68 (79)
Fair	12 (14)
Poor	3 (3.5)
Edentulous gum	16 (18.6)
Dental wearer	9 (10.5)
Tumor characteristics	
Tumor site	
Nasopharynx	37 (43)
Oropharynx	25 (29.1)
Oral cavity	24 (27.9)
T stage	
T1	13 (15.1)
T2	18 (21)
T3	15 (17.4)
T4	40 (46.5)
N stage	
N0	20 (23.3)
N1	19 (22.1)
N2	37 (43)
N3	10 (11.6)
Radiotherapy	
Postoperative RT	28 (32.6)
Radical RT	58 (67.4)
RT technique	
Conventional	37 (43)
IMRT	49 (57)
RT dose	
Median (Gy) (IQR)	70 (66–70)
Range	20–70
Chemotherapy	
Concurrent chemotherapy	67 (78)
No chemotherapy	19 (22)

DM, diabetes mellitus; HT, hypertension; HIV, human immunodeficiency virus infection; IMRT, intensity-modulated radiation therapy

Twenty-eight (32.6%) patients received postoperative RT while 58 (67.4%) patients received definitive RT by a conventional technique in 37 (43%) patients and IMRT technique in 49 (57%) patients. The median RT dose was 70 Gy (range 20–70). Three patients refused to continue their treatment at the 10th, 14th, and 17th fractions, respectively. Sixty-seven (78%) patients received concurrent chemotherapy with the regimen of weekly cisplatin in 32 patients and weekly carboplatin in 35 patients. A dose of 10 cm^3^ of 2% viscous lidocaine was prescribed to 25 patients (29%) four times a day from their 4th to 7th week of RT.

### Incidence of fungal colonization and the accuracy of clinical oral candidiasis

The incidence of fungal colonization and the accuracy of clinical oral candidiasis of each time-period is shown in Table 2. All 86 eligible patients had no clinical diagnosis of oral candidiasis before starting the treatment. A swab was collected at baseline before RT. Fungal colonization from the baseline oral and tongue swab was found in 34 patients. The accuracy of clinical candidiasis pre-treatment was 60%. During the treatment, three patients refused to continue their treatment and one patient refused the oral/tongue swab. Consequently, we had 82 patients remaining for the second swab. Among these, 13 patients had signs and symptoms of clinical oral candidiasis which found at the median RT fraction of 11th (range 7th–14th).

**Table 2:** Accuracy of clinical diagnosis of oral candidiasis

Oral tongue swab	Incidence n (%)	Clinical diagnosis of candidiasis			Accuracy (%)
		Yes	No	Total	
Before RT (n = 86)	Colonized patients	0 (TP)	34 (FN)	34	60
	Non-colonized patients	0 (FP)	52 (TN)	52	
During RT^a^ (n = 82)	Colonized patients	21 (TP)	33 (FN)	54	50
	Non-colonized patients	8 (FP)	20 (TN)	28	
End of RT (n = 71)	Colonized patients	10 (TP)	31 (FN)	41	52
	Non-colonized patients	3 (FP)	27 (TN)	30	

RT, radiotherapy; TP, true positive; TN, true negative; FP, false positive; FN, false negative

^a^15th–17th fraction of RT or immediately following clinical diagnosis of oral candidiasis

Oral/tongue swabs was performed immediately and antifungal agents were prescribed in all cases. The other 69 patients received the second swab halfway through their RT (15th–17th fraction). The confirmatory fungal colonization during RT was positive in 54 patients. Therefore, the accuracy of clinical candidiasis during the treatment was only 50%. At the end of treatment, 71 patients remained on the study. Thirteen patients were diagnosed with oral candidiasis. Fungal colonization at the end of RT was found in 41 patients, subsequently, the accuracy of clinical candidiasis was 52%.

Fungal colonization was most commonly discovered during RT followed by immediately after finishing RT and before starting RT at an incidence of 66%, 58%, and 39%, respectively. We detected 136 colonized specimens in all positive colonized patients and identified ten fungal species. The majority of fungal species were *C. Albicans* in 112 specimens of all collected swabs which were also the most common species found at every period, i.e., before RT, during RT, end of RT. The second most common species was *C. tropicalis*. Table 3 summarizes the fungal species in 136 specimens from all positive fungal colonized patients in each time-period.

**Table 3:** Fungal species in 136 specimens from positive fungal colonized patients in each time-period

Species	Before RT	During RT or at the time of clinical candidiasis	End of RT	Total no. in each species n (%)
*C. albicans*	23	50	39	112 (82.4)
*C. tropicalis*	5	3	0	8 (6)
*C. kursei*	0	0	1	1 (0.7)
*C. grabrata*	1	2	1	4 (2.9)
*Aspergillus flavu*	0	1	0	1 (0.7)
*Penicillium* spp.	1	1	0	2 (1.5)
*Trichosoporon* spp.	2	0	0	2 (1.5)
*Cladosporium* spp.	1	0	0	1 (0.7)
*Exophiala* spp.	0	1	0	1 (0.7)
*Candida* spp.	3	0	1	4 (2.9)
Total no. in each time-period (%)	36 (26.5)	58 (42.6)	42 (30.9)	136

Some culture specimens had more than 1 species in the colonization

### Baseline fungal colonization before RT in all 86 patients

At baseline before starting RT, we found that 39.5% of the patients already had fungal colonization (Table 2). Fungal colonies grew in 41% of male and 37% of female patients before RT which was no different in the non-colonized patients. This study indicated three factors: age (*p* = 0.021), current or previous smoker (*p* = 0.039), and current or previous chewer of betel nuts (*p* = 0.022) were statistically significant different risk factors between colonized and non-colonized patients as shown in Table 4. We could not demonstrate any differences between the two groups in other variables, i.e., underlying disease, the primary tumor site, staging of the tumor, oral hygiene status, and dental wearing. For 34 patients who had positive fungal colonization before starting RT, we found that 11 patients (32.3%) subsequently developed clinical disease. All of them had a history of smoking and two of them continued their smoking during the treatment.

**Table 4:** Fungal colonization at baseline before treatment by characteristics

Factors	Culture at baseline n (%)		*P* value
	Not colonized	Colonized	
Sex			0.815
Male	35 (59)	24 (41)	
Female	17 (63)	10 (37)	
Age, median (IQR)	60 (54–64)	64 (58–70)	0.021*
Underlying disease			
DM	3 (60)	2 (40)	1.000
HT	14 (61)	9 (39)	1.000
HIV	0 (0)	1 (100)	0.395
Tumor site			0.206
Nasopharynx	26 (70)	11 (30)	
Oropharynx	12 (48)	13 (52)	
Oral cavity	14 (58)	10 (42)	
Smoking			0.039**
Never used	17 (81)	4 (19)	
Use/experience	35 (54)	30 (46)	
Alcohol			1.000
Never used	19 (61)	12 (39)	
Use/experience	33 (60)	22 (40)	
Chewing betel			0.022**
Never used	49 (65)	26 (35)	
Use/experience	3 (27)	8 (73)	
Oral status			
Oral hygiene			0.088
Excellent/good	46 (65)	25 (35)	
Fair/poor	6 (40)	9 (60)	
Denture wearer	5 (56)	4 (44)	0.735

*Wilcoxon rank-sum test

**Fisher’s exact test

### Risk factors of fungal colonization throughout the treatment (during and end of RT)

Age, CCRT, dental wearer, 2% viscous lidocaine solution use, xerostomia and mucositis during treatment, and fungal colonization before RT were included in the multivariate analysis (*p* < 0.25). Three factors, receiving CCRT, increasing age, and use of 2% viscous lidocaine solution were independently associated with a higher incidence of fungal colonization throughout the treatment with RT with or without chemotherapy. Adjusted ORs were 4.29 (95% CI 1.12–16.49, *p* = 0.021) inpatient who received CCRT compared to RT alone and 3.62 (95%CI 1.15–11.34, *p* = 0.027) in patients who used 2% viscous lidocaine solution. Adjusted OR increased 1.06 (95% CI 1.01–1.123) every single year of increasing age (Table 5).

**Table 5:** Logistic regression of risk factors of colonization throughout the treatment

Patient characteristics	Univariate		Multivariate	
	OR (95 %CI)	*p* value	OR (95 %CI)	*p* value
Sex				
Male	1			
Female	1.42 (0.49–4.12)	0.523		
Age	1.03 (0.99–1.08)	0.168*	1.06 (1.01–1.123)	0.021^#^
Underlying disease				
DM	0.52 (0.08–3.36)	0.496		
HT	0.78 (0.27–2.23)	0.641		
Tumor site				
Nasopharynx	1			
Oropharynx	1.10 (0.33–3.60)	0.880		
Oral cavity	0.84 (0.26–2.69)	0.770		
Radiotherapy option				
Postoperative RT	1			
Radical RT	1.15 (0.42–3.14)	0.790		
Dose of radiotherapy (Gy)	1.08(1.01–1.16)	0.031*		
CCRT	3.41 (1.16–9.99)	0.026*	4.29 (1.12–16.49)	0.034^#^
CCRT cycle	1.06 (0.64–1.78)	0.820		
Type of chemotherapy agent				
Cisplatin	1			
Carboplatin	1.16 (0.36–3.80)	0.798		
Personal history				
Smoking				
Never used	1			
Using currently	0.82 (0.26–2.56)	0.727		
Alcohol				
Never used	1			
Using urrently	1.54 (0.58–4.09)	0.387		
Oral status				
Oral hygiene				
Excellent/good	1			
Fair/poor	0.68 (0.20–2.25)	0.527		
Denture wearer	0.41 (0.10–1.68)	0.216		
Mouthwash using				
Non-benzydamine HCl	1			
Benzydamine HCl	1.05 (0.35–3.10)	0.934		
2% viscous lidocaine solution using	2.91 (1.03–8.18)	0.043*	3.62 (1.15–11.34)	0.027^#^
Xerostomia	3.51 (0.74–16.70)	0.115*		
Mucositis	2.85 (1.01–8.03)	0.048*		
Candida colonization before RT	4.32 (1.32–14.14)	0.016*		

*p < 0.25, ^#^ significant risk factors

### Risk factors of clinical diagnosis throughout the treatment (during and end of RT)

Female gender, radical RT, current smoker, Benzydamine HCl mouthwash use, 2% viscous lidocaine solution use, xerostomia and mucositis during the treatment, and fungal colonization before RT were included in the multivariate analysis (*p* < 0.25). Only 2 factors; female gender and current smoker were independently associated with a higher incidence of clinical diagnosis of candidiasis throughout the treatment with RT with or without chemotherapy. Adjusted ORs were 10.93 (95% CI 2.26–52.86, *p* = 0.003) in female patients and 12.57 (95%CI 2.19–72.29, *p* = 0.005) in patients who were currently smoking (Table 6).

**Table 6:** Logistic regression of risk factors of clinical candidiasis throughout the treatment

Patient characteristics	Univariate		Multivariate	
	OR (95 %CI)	*p* value	OR (95 %CI)	*p* value
Sex		0.038*	10.93 (2.26–52.86)	0.003^#^
Male	1			
Female	2.71 (1.06–6.95)			
Age	1.02 (0.98–1.07)	0.269		
Underlying disease				
DM	2.45 (0.39–15.52)	0.341		
HT	1.27 (0.48–3.36)	0.629		
Tumor site				
Nasopharynx	1			
Oropharynx	1.16 (0.38–3.55)	0.790		
Oral cavity	3.05 (1.04-9.00)	0.043		
Radiotherapy option				
Postoperative RT				
Radical RT	1 0.51 (0.20–1.29)	0.158*		
Dose of radiotherapy (Gy)	0.90 (0.81–1.01)	0.066*		
CCRT	1.72 (0.55–5.45)	0.354		
CCRT cycle	0.34 (0.54–1.29)	0.419		
Type of chemotherapy agent				
Cisplatin	1			
Carboplatin	0.63 (0.24–1.68)	0.357		
Personal history				
Smoking				
Never used	1			
Using currently	2.56 (0.83–7.85)			
Alcohol				
Never used	1	0.100*		
Using currently	0.68 (0.28–1.67)	0.400	12.57 (2.19–72.29)	0.005^#^
Oral status				
Oral hygiene				
Excellent/good	1			
Fair/poor	0.57 (0.16–1.98)	0.373		
Denture wearer	0.75 (0.17–3.23)	0.700		
Mouthwash using				
Non-benzydamine HCl	1			
Benzydamine HCl	0.44 (0.15–1.28)	0.133*		
2% viscous lidocaine solution using	2.03 (0.74–5.58)	0.172*		
Xerostomia	2.03 (0.72–5.71)	0.180*		
Mucositis	4.13 (1.60-10.69)	0.003*		
Candida colonization before RT	2.13 (0.87–5.21)	0.100*		

*p < 0.25, ^#^significant risk factors

## Discussion

The systematic review by the Multinational Association of Supportive Care in Cancer (MASCC)/International Society of Oral Oncology (ISOO) reported the prevalence of oral candidiasis for all cancer treatments to be 7.5% before starting treatment [17]. This was more than double the increase in comparison to the 3% in our study. We enrolled 97 patients onto the study and 3 patients were excluded due to a clinical diagnosis of oral candidiasis before the study began. MASCC/ISOO also reported that the prevalence of fungal colonization was 48.2% before all cancer treatments [17]. This was comparable to our findings of 39.5%. With an accuracy of clinical candidiasis before treatment at 60%, clinicians need to take into account the fact that oral candidiasis occurs even before any treatment for HNC patients begins, particularly in the patients with significant detrimental risk factors for fungal colonization at baseline, i.e., ex-or current smoker, increasing age, and ex-or current history of betel nut chewing.

MASCC/ISOO reported a weighted prevalence of oral candidiasis during treatment at 39.1% [17] which was in line with the current study at 35.4%. With the focus on the incidence of growth of fungal colonies, 65.9% of patients had confirmed positive cultures for fungal microorganisms in this study. This was slightly lower than a systematic review by the group from MASCC/ISOO [17]. They reported a prevalence of fungal colonization during RT of 74.5%. When considering the clinically diagnosed incidence of oral candidiasis throughout the treatment in this study, the accuracy was as low as 50% and 52% for during and at the end of RT, respectively. Some clinical presentations of erythematous and angular cheilitis forms of candidiasis could be obscured by radiation-induced mucositis. This could be the explanation for the underestimation of clinical diagnosed oral candidiasis during RT. Many studies summarized the clinical appearance of oral candidiasis [12–14]. We suggested careful oral examination during RT focusing on the various types of oral candidiasis. Our study also performed an oral/tongue swab at the end of RT. The fungal colonization at the end of treatment was still high, being found to be the same as during RT at a rate of 57.8%. Jham et al. [5] reported an incidence of colonization of 71% at the end of RT. Ramirez-Amador et al. [6] reported the prevalence of positive candida cultures at 62% at completion of RT. These findings were meaningful for the clinicians, highlighting the need to pay close attention and prescribe the necessary interventions at the end of treatment.

In non-pathological circumstances, the most common characteristic oral flora consists of Candida species. Under some physiological and pathological conditions, Candida may change status from that of commensalism to a pathogen [3, 5]. *Candida albicans* has been identified as being the most frequent species in head and cancer patients in most studies [3–5, 7, 8]. Our results are similar showing that *C. albicans* was the most commonly found species, being as high as 82% throughout the treatment, the second and third most common being non-albicans species; *C. tropicalis*, and *C. glabrata*.

Previous studies have reported controversial risk factors of oral candidiasis and colonization in HNC patients receiving RT [5, 6, 9, 18]. Focusing on the clinical diagnosis of candidiasis throughout the treatment, smoking and being female were the independent factors in our study, findings in line with the other research [5, 9]. Epstein et al. [9] found the presence of oral prosthesis, alcohol use, and smoking represent risk factors for oral colonization. Panghal et al. demonstrated that Grade 4 mucositis is the most significant risk factor of oral candida infection [18]. Some studies demonstrated the correlation between xerostomia and the risk of oropharyngeal infection [5, 6]. These findings were in contrast to our study. Even though significance in the univariate analysis was demonstrated with radiation-induced oral mucositis and xerostomia during the treatment, both acute side effects were not significant in the multivariate analysis for the fungal colonization throughout the treatment. Ramirez-Amador et al. reported that smoking and denture wearing were not risk-factors for increased fungal colonization [6]. In this study, we found that increasing age, smoking, and chewing betel nuts were associated with fungal colonization at baseline before the treatment, although throughout the treatment, three factors were significantly related to fungal colonization: the addition of chemotherapy into RT, increasing age, and using a 2% viscous lidocaine solution. RT itself damages the local oral mucosa and salivary glands. These injuries lead to an increase in fungal colonization. CCRT with cisplatin has been found to synergistically induce severe mucosal injury [19]. Increasing age has been reported as one of the risk factors of fungal colonization in many studies [3, 5, 8, 18] and our result were consistent with their findings. It was surprising that using a 2% viscous lidocaine solution was another risk factor for fungal colonization in our study. Three in-vitro studies [20–22] supported the efficacy of lidocaine as an antifungal drug. The first study [21] found a dose-dependent fungicidal effect of lidocaine. Another study demonstrated the in-vitro effect of lidocaine on *C. albicans* germ tube formation [22]. Palmeira-de-Oliveira et al. [20] reported the dose-dependent fungicidal effect of lidocaine with the concentrations varying from 15 to 30 30 mg/ml. They also noticed that different species of candida had different susceptibility to this drug, i.e., *C. krusei* and *C. albicans* ATCC10231 responded to lidocaine at a minimum inhibitory concentration (MIC) of 10 mg/mL while other *C. albicans* needed an increasing concentration [20]. Although we did not use the commercial preparation of 2% viscous lidocaine solution in this study the concentration of the hospital preparation that we prescribed for our patients was at least higher than the MIC to which Candida was susceptible. However, we did not record the level of patient adherence to all prescribed drugs during the treatment including this solution. In view of this, caution must be paid to this finding and we could not strongly conclude that lidocaine solution is one of the risk factors associated with fungal colonization.

As far as we know, this is the first reported study on the accuracy of clinically diagnosed oral candidiasis by confirmation with fungal colonization in HNC receiving both RT alone and CCRT. We tried to analyze all determinants which could be related to fungal colonization before and throughout the treatment, e.g., patient factors, treatment factors, and treatment complications.

There are also some limitations to this study. First, some patients dropped out and did not receive oral/tongue swabs during and at the end of treatment. Second, Benzydamine HCl and 2% viscous lidocaine solution were used as analgesic drugs for treating radiation-induced mucositis in the patients which may have impacted on the data. According to an in-vitro study [21], both local anesthetics showed a supplemental benefit in the treatment of candidiasis. Patients who used either or both drugs might not represent the true rate of fungal colonization during and at the end of RT/CCRT. Third, the levels of patient adherence to 2% viscous lidocaine solution during the treatment were not recorded. Consequently, this study has some inconsistencies in adding weight to the fungicidal effects described in some previous studies [20–22]. Hence, the results of 2% viscous lidocaine solution as one of the risk factors in this study should be interpreted with caution. Forth, we prescribed anti-fungal agents immediately for every patient who had only clinically diagnosed oral candidiasis during the treatment, which could not represent an accurate rate of fungal colonization during RT/CCRT and at the end of treatment.

## Conclusions

Our findings demonstrated the underestimation of oral candidiasis using clinical signs and symptoms in HNC patients before and throughout RT and CCRT. We do not suggest the performing of oral and tongue swabs result in higher accuracy, but crucial attention to clinical signs in the oral examination could improve decision making for the detection of oral candidiasis. Diagnosis is still regularly made by clinical findings, however, fungal culture may be necessitated and useful, particularly in patients with identifiable risk factors.

## Data Availability

The datasets used and/or analyzed during the current study are available from the corresponding author on reasonable request.

## Abbreviations

HNC: Head and neck cancer
RT: Radiotherapy
CCRT: Concurrent chemoradiotherapy
